# Editorial

**Published:** 1877-02

**Authors:** 


### Editorial.

As the news of the misfortune which has overtaken the publishing house of W. B. Keen, Cooke & Co. will reach the readers of the MEDICAL JOURNAL AND EXAMINER before the February number will make its appearance, and may cause doubt on the part of the subscribers as to our future, we take occasion to say that the JOURNAL AND EXAMINER will be issued with its accustomed regularity.

The Medical Press Association is pledged for its success, and will see that under no circumstances will it be allowed to fail, either in its promptitude or excellence.

We cannot now inform our readers just what arrangements for the publication of the JOURNAL AND EXAMINER will be made, but we can assure them that it will be published. In our next issue we hope to be able to give them every information in reference to our plans and prospects.

We deplore the disastrous effects hard times have wrought upon our friends and publishers, heartily sympathize with them in their trials, and hope that very soon they will recover from them and become more prosperous than ever.

We will close this editorial note by expressing our warm appreciation of the generous and diligent co-operation we have received at their hands, in our efforts to make the MEDICAL JOURNAL AND EXAMINER a first-class medical periodical.

☞ Until futher notice all Subscriptions and Communications should be sent to the EDITOR, at the office of publicatlon, 115 State Street.

